# Assessment of efficacy in automated plan generation for Varian Ethos intelligent optimization engine

**DOI:** 10.1002/acm2.13539

**Published:** 2022-01-27

**Authors:** Shyam Pokharel, Abilio Pacheco, Suzanne Tanner

**Affiliations:** ^1^ Department of Radiation Oncology GenesisCare Naples Florida USA; ^2^ Department of Radiation Oncology Boca Raton Regional Hospital Baptist Health South Florida Lynn Cancer Institute Boca Raton Florida USA

**Keywords:** adaptive radiotherapy, auto planning, intelligent optimization engine, prostate cancer, Varian Ethos

## Abstract

Varian Ethos, a new treatment platform, is capable of automatically generating treatment plans for initial planning and for online, adaptive planning, using an intelligent optimization engine (IOE). The primary purpose of this study is to assess the efficacy of Varian Ethos IOE for auto‐planning and intercompare different treatment modalities within the Ethos treatment planning system (TPS). A total of 36 retrospective prostate and proximal seminal vesicles cases were selected for this study. The prescription dose was 50.4 Gy in 28 fractions to the proximal seminal vesicles, with a simultaneous integrated boost of 70 Gy to the prostate gland. Based on RT intent, three treatment plans were auto‐generated in the Ethos TPS and were exported to the Eclipse TPS for intercomparison with the Eclipse treatment plan. When normalized for the same planning target volume (PTV) coverage, Ethos plans *D*_max%_ were 108.1 ± 1.2%, 108.4 ± 1.6%, and 109.6 ± 2.0%, for the 9‐field IMRT, 12‐field IMRT, and 2‐full arc VMAT plans, respectively. This compared well with Eclipse plan *D*_max%_ values, which was 108.8 ± 1.4%. OAR indices were also evaluated for Ethos plans using Radiation Therapy Oncology Group report 0415 as a guide and were found to be comparable to each other and the Eclipse plans. While all Ethos plans were comparable, we found that, in general, the Ethos 12‐field IMRT plans met most of the dosimetric goals for treatment. Also, Ethos IOE consistently generated dosimetrically hotter VMAT plans versus IMRT plans. On average, Ethos TPS took 13 min to generate 2‐full arc VMAT plans, compared to 5 min for 12‐field IMRT plans. Varian Ethos TPS can generate multiple treatment plans in an efficient time frame and the quality of the plans could be deemed clinically acceptable when compared to manually generated treatment plans.

## 1 INTRODUCTION

Radiation therapy is a constantly changing and evolving field.^1^, ^2^, ^3^, ^4^, ^5^, ^6^ There has been a significant improvement in radiation therapy treatment planning, optimization, and delivery in the last decade.^3^, ^4^, ^5^ Several techniques have been developed to optimize overall treatment planning and delivery to increase therapeutic gain.^4^, ^5^, ^6^, ^7^, ^8^, ^9^, ^10^, ^11^, ^12^, ^13^, ^14^, ^15^ Lately, online adaptive therapy with daily treatment planning and delivery is being introduced into the radiation therapy community.^16^, ^17^, ^18^, ^19^, ^20^ However, the challenge for such a system is to create a clinically acceptable plan in an efficient timeframe. Treatment planning is a multi‐step, iterative process with several, mutually conflicting objectives.^5^, ^6^ Historically, a treatment plan is manually generated by a planner, using tools available to them in the treatment planning system (TPS). In a trial‐and‐error fashion, the planner iteratively adjusts different planning objectives with the goal of creating a clinically acceptable treatment plan that meets most, if not all, of the objectives in a reasonable amount of time. The quality of the plan varies between individual planners and institutional protocols.^12^ To create a consistent and optimal plan, independent of the planner's skills, several investigators have explored different techniques to create plans automatically.^1^, ^2^, ^3^, ^4^, ^5^, ^6^

Currently, there are two, commercially available automated treatment planning techniques that have been extensively evaluated.^6^, ^7^, ^8^, ^9^, ^10^, ^11^, ^12^, ^13^ One technique is an algorithm‐based optimization engine that mimics the real planner by iteratively adjusting the plan objective function to create a clinically acceptable plan. The other technique, known as knowledge‐based planning (KBP), uses machine‐learning algorithms to mine for desirable planning metrics within an institution's database and builds a model for new patient plans. Both techniques have shown to create acceptable plans while minimizing planner variability. Pinnacle (Fitchburg, Wisconsin, USA) auto‐planning (AP) is an example of the first technique and Varian (Palo Alto, California, USA) Eclipse RapidPlan (RP) is an example of the second technique.^6^, ^7^ Several groups have investigated Pinnacle AP and Eclipse RP for different anatomical sites.^6^, ^7^, ^8^, ^9^, ^10^, ^11^, ^12^, ^13^ The overall conclusion of these investigations was that these automated treatment planning techniques are capable of creating equivalent plans to that manual plans created by an experienced planner. Smith et al. demonstrated that both Pinnacle AP and Eclipse RP create plans with similar dosimetric qualities. Similarly, Cornell et al. demonstrated that Eclipse RP plans offered clinically acceptable plans, while reducing the planning metric variability between plans. Although KBP further facilitates the treatment planning process while maintaining clinical quality, it is limited to the number and quality of treatment plans in the database, which stem from one institution. Several groups have also investigated the feasibility of artificial intelligence (AI)‐based automated planning.^21^, ^22^, ^23^, ^24^, ^25^, ^26^ Currently, one TPS Vendor (RaySearch Laboratories, Stockholm, Sweden) provides AI‐based automated treatment planning.^27^ AI‐based auto‐segmentation is available for either a reference CT or online daily cone‐beam computed tomography (CBCT) scans to create an adaptive treatment plan.^16^, ^28^

Varian has recently released a new adaptive treatment planning and delivery system called Ethos.^16^, ^17^, ^18^, ^19^ The Ethos system was built on a Varian Halcyon platform with several added features for automated planning, adaptive planning for treatment delivery, and treatment monitoring with a dedicated server. The treatment delivery console makes use of a synthetic CT, which is the planning CT that is deformably registered to the adaptive treatment session's CBCT. AI‐based auto‐segmentation structures are then generated for an adaptive treatment plan, which can then be reviewed and approved by a clinician. Next, a dose calculation is performed onto the synthetic CT and an adaptive plan is finalized prior to clinical use. This general process would be executed for each adaptive treatment session.

Ethos uses an intelligent optimization engine (IOE) to generate plans automatically. IOE is an algorithm that mimics the real treatment planner by setting up the optimization objective function for the photon optimization (PO) algorithm and then controls and monitors the optimization process. IOE converts different clinical goals for both targets and organs at risk (OAR) from radiation therapy (RT) intent into the optimization objective function. RT intent is the prescription interface in Ethos, where users must rank the clinical goals from 1 to 5: 1, most important; 2, very important; 3, important; 4, less important; 5, report value only. Also, the physician defines what he/she intends to treat and establishes dose‐fractionation regimens in this interface, thus becoming the planning directive in Ethos. During automated planning, the IOE algorithm can create optimization “helper” structures, such as dose rings for target structures. IOE can also add extra “helper” objectives to control plan quality metrics, such as plan monitor units (MU) and normal tissue objectives. In instances where structures with conflicting goals overlap, IOE can create new, non‐overlapping optimization structures. This aids the planning process by controlling the doses to the respective structures and still achieve planning goals, much like a skilled planner would address these issues in a manual planning process. Once the planner defines clinical importance, the IOE handles the process of reassigning weights for different objectives. Site‐specific dose‐fractionation regimens, clinical goals, and planning objectives can be preset by generating plan templates. The primary purpose of this study is to assess the efficacy of Varian Ethos IOE for automated planning of prostate cancer treatment. A secondary purpose of this study is to evaluate and intercompare different treatment modalities within the Ethos TPS.

## 2 METHODS AND MATERIALS

A total of 36 retrospective prostate cases, treated at our institution between July 2020 and October 2021, were selected for this study. Specifically, all cases were planned for the prostate and proximal seminal vesicles with a dose of 50.4 Gy in 28 fractions to the proximal seminal vesicles and a simultaneous integrated boost (SIB) to the prostate gland, with a dose regimen of 70 Gy in 28 fractions. This dose regimen corresponds to Arm 2 of the Radiation Therapy Oncology Group (RTOG) report 0415, which was used as a general guide for planning objectives.^29^ Table 1 provides an overview of RTOG 0415 target and normal tissue guidelines for Arm 2 of the protocol. Fifteen cases were originally planned on the Eclipse TPS and then exported to Ethos for auto‐plan generation based on RT intent templates, while the remaining 21 cases were originally planned on the Ethos TPS (v.2.00.10) and exported to the Eclipse TPS for manual plan generation. Treatment plans were generated by three certified dosimetrists and were clinically approved by radiation oncologists prior to treatment. All cases were manually planned on the Varian Eclipse TPS (v.16.1.0) and planned for the Halcyon linear accelerator, which is an identical machine to the Ethos linear accelerator and uses the same gold‐beam data in the TPS. Eclipse PO algorithm was used for plan optimization and Acuros XB algorithm (AXB) was used for final volumetric dose calculation with a 0.2 cm calculation resolution. Approved treatment plans were normalized such that PTV coverage was between 95% and 99%.

**TABLE 1:** RTOG 0415 target and normal tissue guidelines

**Dose goal (Arm 2)**	**Minimum PTV dose (encompassing ≥ 98% of PTV)**	**Minimum CTV dose (encompassing ≥ 100% of CTV)**	**Maximum dose to PTV (no variation)**	**Maximum PTV dose to PTV (minor variation)**	**Maximum PTV dose to PTV (major variation)**
	70 Gy	70 Gy	74.9 Gy	**77 Gy**	**>77 Gy**

**Normal organ limit (Arm 2)**	**No more than 15% receives dose that exceeds**	**No more than 25% receives dose that exceeds**	**No more than 35% receives dose that exceeds**	**No more than 50% receives dose that exceeds**	
Bladder constraint	79 Gy	74 Gy	69 Gy	64 Gy	
Rectum constraint	74 Gy	69 Gy	64 Gy	59 Gy	
Penile bulb	Mean dose less than or equal to 51 Gy (72.8% of PD)				

For treatment plans that originated in the Eclipse TPS, the CT datasets and structure sets were imported into the Ethos TPS and a pre‐generated RT intent template was selected for plan generation. These templates specify dose‐fractionation regimen and derived structures, which are automatically generated structures such as margins for PTV. Once imported into Ethos, the RT intent was confirmed to match that of the Eclipse TPS. The RT intent in Ethos is the equivalent to the prescription in Eclipse TPS. Therefore, we needed to ensure that the correct RT intent was applied to the Ethos plan generation. Next, each structure in the Eclipse structure set was matched to its Ethos structure set counterpart. If an Eclipse structure did not have an Ethos counterpart, a structure was added to the Ethos template and was then manually matched. Finally, derived structures were automatically generated, based on user‐defined rules. User‐defined rules relate to a particular clinic's approach to generating derived structures. Examples of derived structures could be margins for PTV, applying Boolean operators for prostate and seminal vesicles, and so forth. Derived structures are defined under the template management interface of Ethos TPS.

After contours were generated, technical structures were set and confirmed. The technical structures module includes the simulation isocenter location, couch plane location, and density correction structures. Simulation isocenter and couch plane location were visually confirmed for proper placement. Automated density correction structures were generated for setup markers on the surface of the body and internal markers. On occasion, Ethos would automatically set the setup markers to a titanium mass density and would thus need to be revised and manually set to air. The current version of Ethos does not auto‐generate bladder and/or rectal contrast in the technical structures module. Also, the current version of Ethos does not allow for the import of density‐overridden structures within its technical structures interface, even though these structures were generated, and a density override value was applied in the Eclipse TPS. As a result, structures with a density‐override were manually generated, matching the volume in the Eclipse TPS as best as possible.

Once all contours and technical structures were imported and generated, the Dose Preview module was selected. As mentioned elsewhere,^16^, ^17^, ^18^, ^19^ the Dose Preview module uses the IOE, PO, and Fourier Transform Dose Calculation algorithm to auto‐generate a dose fluence based on user‐defined goals, using a representative set of beam data and closely approximating a 9‐field IMRT plan. After several seconds, the Dose Preview module displayed the results of the dose fluence via 3D dose representation, dose‐volume histograms (DVH), and achieved treatment planning goals. If any given treatment planning goal is not met, the user has the option to refine the preliminary plan by re‐ordering the planning goals or adding new goals to the template. However, to assess the efficacy of the Ethos TPS and its auto‐generated plans, all plans were authorized without any intervention or manipulation. Currently, Ethos TPS does not have the functionality to create RP models, though the user has the option to import preconfigured RP models from Eclipse into Ethos TPS. RP models can then be incorporated into the planning directive and add additional objectives in the planning optimization process. However, RP models were not utilized in the planning optimization process in this study.

**FIGURE 1:**
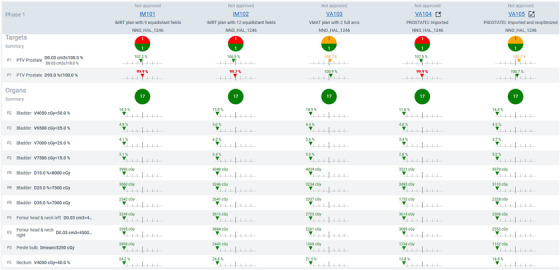
Plan selection interface of Ethos treatment planning system (TPS), displaying all available plans for plan approval with a summary of PTV and organs at risk (OAR) dose metrics and a visual queue if the metrics were met. IM101 = 9‐field IMRT, IM102 = 12‐field IMRT, VA103 = 2‐field VMAT, VA104 = 2‐field VMAT Eclipse plan import, VA105 = 2‐field VMAT Eclipse plan import and re‐optimized

After the plans were authorized, the Ethos TPS utilized the IOE to auto‐generate plans in the Plan Review module. Ethos can generate up to five plans for a centrally located target: 7‐field IMRT, 9‐field IMRT, 12‐field IMRT, 2‐full arc VMAT, and 3‐full arc VMAT. In addition, Ethos generates two additional plans for laterally located targets: 7‐field lateral IMRT and 2‐partial arc VMAT. Furthermore, Ethos TPS can apply a plan normalization value to meet a desired target volume coverage. Our clinic preset Ethos to auto‐generate three plans: 9‐field IMRT, 12‐field IMRT, and 2‐full arc VMAT plans for centrally located targets, without a plan normalization value. In addition to the auto‐generated plans, the Eclipse treatment plan was directly imported into the Ethos TPS via the Plan Review module. In doing so, Ethos generated two additional plans: Imported and Imported and reoptimized plans. **Figure** **1** shows dosimetric indices tabulated for each Ethos plan and highlights indices that are out of tolerance. Ethos TPS used the AXB algorithm for final volumetric dose calculation with the calculation resolution set in configuration settings to 0.25 cm. Once all plans were generated, planning time was assessed to determine the duration of plan generation for each plan candidate. All plans were exported back to the Eclipse TPS for plan and DVH comparisons and dosimetric tabulation. The current version of Ethos does not have a module that allows DVH overlay and comparison for different plans. For treatment plans that originated in the Ethos TPS, all plans were exported to Eclipse. From Eclipse, a planner manually generated a 2‐full arc VMAT treatment plan that best met the RTOG 0415 guidelines. Once generated, dosimetric data were tabulated for comparison with the Ethos plans.

The stochastic nature of IOE was evaluated by performing multiple optimizations for the same planning directive. For the same anatomy, structure set, and planning directive, the IOE provided similar, but not identical, results. Quality indices were recorded for the 9‐field IMRT, 12‐field IMRT, and 2‐full arc VMAT plans and compared with the first optimization Ethos baseline plan. Although results fluctuated, they were not significant enough to change the acceptability criteria as in the baseline plan. The plans included in this study were from the first optimization.

Once imported into the Eclipse TPS, a copy of each Ethos plan was generated. The copied plan was re‐normalized to match the planning normalization value of the Eclipse plan. This allowed for an assessment of the re‐normalized Ethos plan versus the Eclipse plan, where the normalization value was a fixed variable. The un‐normalized Ethos plans were compared to their respective Eclipse plans to determine the efficacy of the Ethos TPS. Using RTOG 0415 as a guide, the following dosimetric quality indices were tabulated to compare treatment plans:

1. PTV V_100%_: Percentage of PTV that receives 100% of the prescription dose (PD).
2. D_max%_: Global maximum dose; tabulated as a percentage of PD.
3. CTV V_100%_: Percentage of CTV that receives 100% of the PD; CTV is defined as the prostate contour, delineated by the radiation oncologist.
4. Bladder V_79Gy_, V_74Gy_, V_69Gy_, and V_64Gy_: Volume of bladder receiving 79, 74, 69, and 64 Gy, respectively.
5. Rectum V_74Gy_, V_69Gy_, V_64Gy_, and V_59Gy_: Volume of rectum receiving 74, 69, 64, and 59 Gy, respectively.
6. Mean Penile Bulb dose; tabulated as a percentage of PD.

In addition to RTOG 0415 indices, mean and maximum doses were tabulated for the bladder and rectum.

Once the Ethos plans were imported into Eclipse, a radiation oncologist reviewed a side‐by‐side plan comparison (including DVH, isodose line distribution, and dosimetric indices) and would determine if the Ethos plans were comparable to the Eclipse plans and, thus, deem them clinically acceptable.

To facilitate with data gathering, third‐party scripting software (ClearCheck, v.1.7.11, Radformation)^30^ was used. Once all the data were tabulated, average and standard deviation values were calculated. Finally, a student pair *t*‐test at 5% level of significance was used to make a statistical comparison of dosimetric quality indices for different Ethos treatment plans, using the Eclipse plan as a reference.

## 3 RESULTS

Once the dose preview was authorized in the Ethos TPS, it took, on average, 2.5, 5, and 13 min to generate the 9‐field IMRT, 12‐field IMRT, and 2‐full arc VMAT plans, respectively. When Ethos plans were exported to Eclipse TPS, the OAR structures were resampled within 0.14cc of the originally contoured structure in the Eclipse TPS. Relative to the original Eclipse contour, resampling variation was −0.6cc to 6.9cc (−0.7% to 8.2%) for the rectum, −0.3cc to 0.5cc (−0.2% to 0.3%) for the bladder, and 0.0cc to 0.03cc (0.0% to 21.4%) for the penile bulb. PTV structures were resampled within 1.4cc of the Eclipse structure (range: −1.8cc to 4.5cc; −1.3% to 3.3%).

**FIGURE 2:**
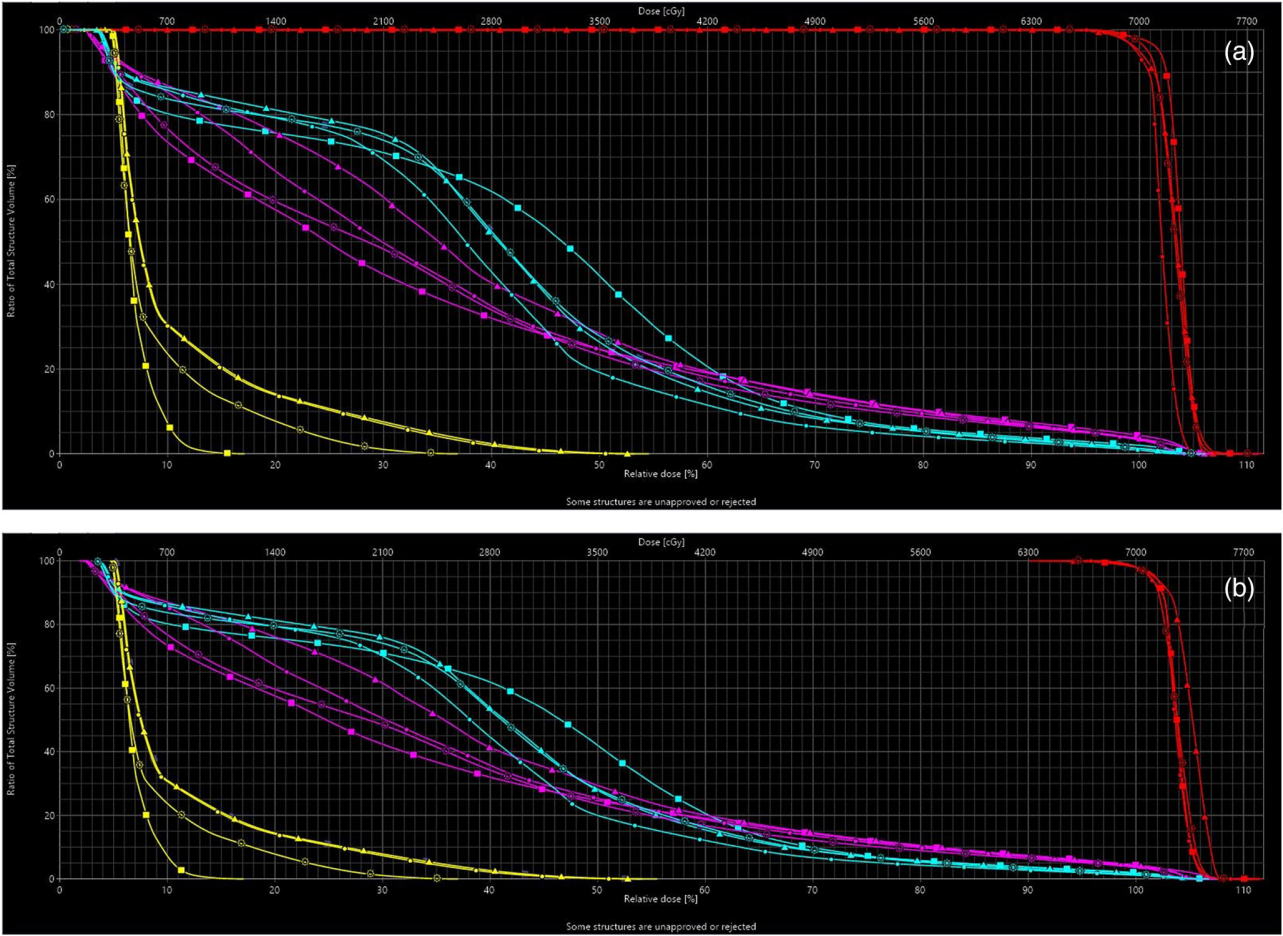
DVH comparison of Eclipse with un‐normalized **(a)** and normalized **(b)** Ethos plans for PTV, bladder, rectum, and bowel. PTV = red, Rectum = cyan, Bladder = magenta, Penile Bulb = yellow. Eclipse plan (‐ ■ ‐), Ethos 9‐field IMRT plan (‐ ▲ ‐), Ethos 12‐field IMRT plan (‐ ● ‐), and Ethos 2‐full arc VMAT (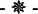)

Un‐normalized Ethos plans displayed comparable dosimetric results, relative to the Eclipse plans. Table 2 shows pertinent dosimetric information for the un‐normalized plans. On average, Ethos total plan MUs were higher than Eclipse plan MUs by 142, 265, 180, and 106 for the 9‐field IMRT, 12‐field IMRT, 2‐full arc VMAT, and 2‐full arc VMAT “Eclipse reoptimized” plans, respectively. On average, PTV coverage for the Ethos plans differed from the Eclipse plans by about −1.3%, −1.9%, 0.3%, and 0.3% for the 9‐field IMRT, 12‐field IMRT, 2‐full arc VMAT, and 2‐full arc VMAT “Eclipse reoptimized” plans, respectively. Variation in PTV coverage for the different Ethos plans were as follows: 93.9%–97.4%, 91.4%–96.8%, 95.1%–98.1%, and 95.5%–98.0% for the 9‐field IMRT, 12‐field IMRT, 2‐full arc VMAT, and 2‐full arc VMAT “Eclipse reoptimized” plans, respectively. Based on these data, the 9‐field and 12‐field IMRT plans needed the most plan normalization. Eclipse plans were normalized such that PTV coverage ranged between 95% and 99.0%. OAR dosimetric indices were also compared for the bladder, rectum, and penile bulb on all Ethos and Eclipse plans. Both Ethos and Eclipse TPS were able to achieve RTOG 0415 OAR objectives for all cases in this study. Although these plans are un‐normalized, one can also see in Table 2 that the OAR maximum doses had slight variations between the different plans. Figure 2a shows a DVH comparison between an Eclipse VMAT plan and un‐normalized 9‐field IMRT, 12‐field IMRT, and 2‐full arc VMAT Ethos plans for one case. In the figure, PTV, rectum, bladder, and penile bulb DVHs are colored red, cyan, magenta, and yellow, respectively.

**TABLE 2:** Comparison of average dosimetric indices for un‐normalized Ethos and Eclipse plans

	**Ethos**								**Eclipse**	
	**9‐fld IMRT**	**σ**	**12‐fld IMRT**	**σ**	**2‐fld VMAT**	**σ**	**Ecl. reopt. 2‐fld VMAT**	**σ**	**2‐fld VMAT**	**σ**
Total plan MUs	1196.5	131.7	1319.1	158.1	1234	124.5	1160.5	98.7	1054.4	170.6
PTV *V*_100%_ (%)	95.5	0.8	94.9	0.9	97.1	0.6	97.0	0.5	96.8	1.3
*D*_max%_	107.4	0.9	107.5	1.5	109.7	1.8	110.1	1.7	108.8	1.4
CTV *V*_100%_ (%)	99.9	0.4	99.9	0.3	99.9	0.3	99.9	0.2	99.5	0.8
Bladder *V*_79Gy_ (%)	0.0	0.0	0.0	0.0	0.0	0.0	0.0	0.0	0.0	0.0
Bladder *V*_74Gy_ (%)	0.0	0.1	0.0	0.1	0.3	0.6	0.6	1.1	0.4	1.1
Bladder *V*_69Gy_ (%)	4.4	2.9	4.2	2.8	4.6	3.4	4.6	3.3	4.6	3.8
Bladder *V*_64Gy_ (%)	6.8	3.9	6.5	3.8	6.6	4.4	6.5	4.3	6.8	5.0
Rectum *V*_74Gy_ (%)	0.0	0.0	0.0	0.0	0.0	0.2	0.0	0.1	0.0	0.1
Rectum *V*_69Gy_ (%)	1.5	1.4	1.5	1.4	1.8	1.7	1.7	1.6	2.0	1.8
Rectum *V*_64Gy_ (%)	3.2	2.5	3.0	2.4	3.3	2.6	3.2	2.6	3.5	2.9
Rectum *V*_59Gy_ (%)	4.9	3.4	4.6	3.4	4.9	3.5	4.8	3.5	5.0	3.9
Penile bulb mean dose (%)	30.5	13.6	30.4	13.9	27.4	13.3	26.2	13.6	19.4	12.1
Bladder max dose (%)	106.0	1.0	106.0	0.9	108.1	1.4	108.4	1.5	107.2	1.6
Rectum max dose (%)	100.2	8.5	100.1	8.0	101.7	9.0	101.9	9.4	102.2	8.5

Abbreviations: σ, standard deviation; 12‐fld IMRT, 12‐field IMRT plan; 2‐fld VMAT, 2‐full arc VMAT plan; 9‐fld IMRT, 9‐field IMRT plan; Ecl. reopt. 2‐fld VMAT, Eclipse re‐optimized 2‐field VMAT plan; PTV, planning target volume; *V*_79Gy_, *V*_74Gy_, *V*_69Gy_, *V*_64Gy_, and *V*_59Gy,_ volume receiving 79, 74, 69, 64, and 59 Gy, respectively.

**FIGURE 3:**
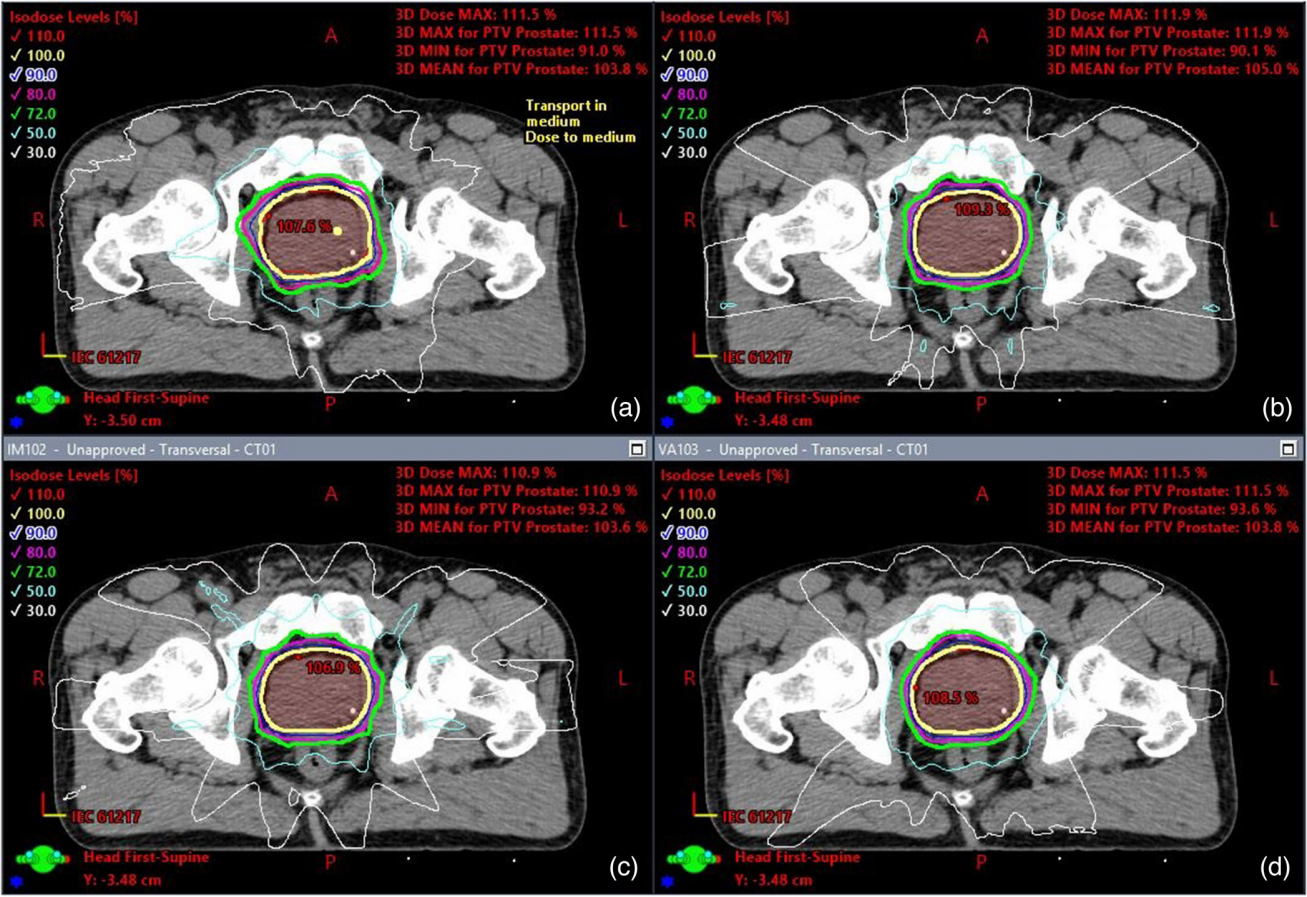
Graphical display of various plans: (a) Eclipse 2‐field VMAT plan, (b) Ethos 9‐field IMRT plan, (c) Ethos 12‐field IMRT plan, (d) Ethos 2‐field VMAT plan. Rx isoline = 100% (bold yellow isoline)

**FIGURE 4:**
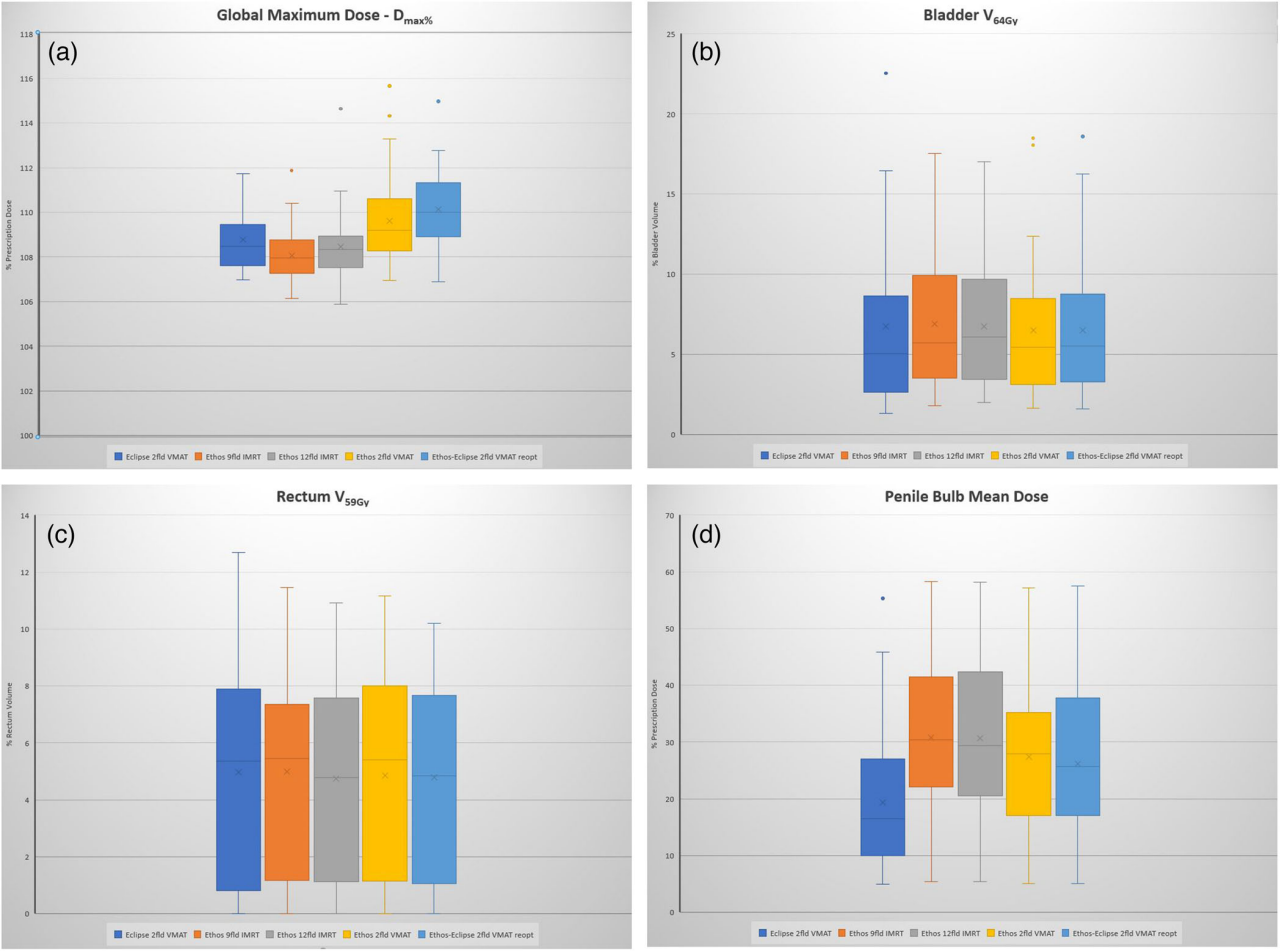
Box and whiskers plot of *D*_max%_ (a), bladder V_64Gy_ (b), rectum V_59Gy_ (c), and penile bulb mean dose (d)

Isodose distributions and DVHs were also compared for the normalized Ethos plans. Figure 3 shows the isodose distribution comparison for one case, displaying the same axial slice for the 2‐full arc VMAT Eclipse plan and the normalized 9‐field IMRT, 12‐field IMRT, and 2‐full arc VMAT plans, respectively. Also, Figure 2b shows a DVH comparison between an Eclipse VMAT plan and normalized 9‐field IMRT, 12‐field IMRT and 2‐full arc VMAT Ethos plans for the same case as in Figure 2a. Although the figures are limited to one case, one can see that the isodose distribution is similar to the Eclipse plan dose distribution and that the Ethos DVHs for the PTV and OARs did not deviate significantly once they were normalized to match the Eclipse plan normalization. Table 3 shows pertinent dosimetric information for the normalized plans, along with their associated *p*‐values. After normalization, *D*_max%_ was 108.8%, 108.1%, 108.4%, 109.6%, and 110.1% for the 2‐full arc Eclipse plan, 9‐field IMRT, 12‐field IMRT, 2‐full arc VMAT Ethos plans, and 2‐full arc VMAT “Eclipse reoptimized” plans, respectively. Compared to the un‐normalized plans, the average change in *D*_max%_ was 0.7%, 0.9%, −0.1%, and 0.01% for the 9‐field IMRT, 12‐field IMRT, 2‐full arc VMAT Ethos plans, and 2‐full arc VMAT “Eclipse reoptimized” plans, respectively. This demonstrates that plan renormalization had a negligible effect on *D*_max%_. Ethos VMAT plans were consistently hotter than the Eclipse VMAT plans (*p* = 0.03 and 0.0001 for 2‐full arc VMAT and 2‐full arc VMAT “Eclipse reoptimized” plans, respectively) and were hotter than the 9‐field IMRT and 12‐field IMRT plans. All Ethos plans more closely met the CTV V_100%_ guideline (range 99.8% to 99.9%) compared to the reference Eclipse plans, which on average was 99.5% coverage. OAR dosimetric indices were relatively unaffected by the plan normalization and despite the minor variations among the dosimetric indices, all Ethos plans continued to meet RTOG 0415 criterion for all OARs and were comparable to the Eclipse plans. For example, Bladder V_64Gy_ was 6.4%, 6.9%, 6.8%, 6.5%, and 6.5% for the 2‐full arc Eclipse plan, 9‐field IMRT, 12‐field IMRT, 2‐full arc VMAT Ethos plans, and 2‐full arc VMAT “Eclipse reoptimized” plans, respectively. Also, Rectum V_59Gy_ was 5.0%, 5.0%, 4.7%, 4.9%, and 4.8% for the 2‐full arc Eclipse plan, 9‐field IMRT, 12‐field IMRT, 2‐full arc VMAT Ethos plans, and 2‐full arc VMAT “Eclipse reoptimized” plans, respectively. Also, it was observed that the Penile Bulb mean dose was lowest for the Eclipse plans (19.4% of PD) when compared to the Ethos plans (30.8%, 30.6%, 27.4%, and 26.2% of PD for the 9‐field IMRT, 12‐field IMRT, 2‐full arc VMAT Ethos plans, and 2‐full arc VMAT “Eclipse reoptimized” plans, respectively). Although the Penile Bulb mean dose was relatively high, all plans still met RTOG guideline (mean dose <51 Gy or 72.8% of PD). Figure 4 shows box plots for *D*_max%_ (a), Bladder V_64Gy_ (b), Rectum V_59Gy_ (c), and Penile Bulb mean dose (d) to display data point distribution for all cases analyzed in this study. All Ethos plans were also deemed comparable to the Eclipse plans and were deemed clinically acceptable by the radiation oncologist.

**TABLE 3:** Comparison of dosimetric indices for normalized Ethos and Eclipse plans. PTV coverage in the Ethos plans match their Eclipse counterpart. *p*‐Value is calculated using Eclipse plan as a reference

	**Ethos**												**Eclipse**	
	**9‐fld IMRT**	**σ**	***p*‐Value**	**12‐fld IMRT**	**σ**	***p*‐Value**	**2‐fld VMAT**	**σ**	***p*‐Value**	**Ecl. reopt. 2‐fld VMAT**	**σ**	***p*‐Value**	**2‐fld VMAT**	**σ**
*D*_max%_	108.1	1.2	0.01	108.4	1.6	0.29	109.6	2.0	0.03	110.1	1.7	≤ 0.001	108.8	1.4
CTV *V*_100%_ (%)	99.9	0.3	0.01	99.9	0.3	0.01	99.8	0.4	0.03	99.8	0.3	0.05	99.5	0.8
Bladder *V*_79Gy_ (%)	0.0	0.0	N/A	0.0	0.0	N/A	0.0	0.0	N/A	0.0	0.0	N/A	0.0	0.0
Bladder *V*_74Gy_ (%)	0.2	0.4	0.47	0.3	0.5	0.60	0.3	0.7	0.89	0.6	1.1	0.36	0.4	1.1
Bladder *V*_69Gy_ (%)	4.6	2.9	0.88	4.5	2.9	0.65	4.6	3.3	0.73	4.6	3.3	0.80	4.6	3.8
Bladder *V*_64Gy_ (%)	6.9	3.9	0.59	6.8	3.9	0.98	6.5	4.4	0.34	6.5	4.2	0.33	6.8	5.0
Rectum *V*_74Gy_ (%)	0.0	0.2	0.67	0.0	0.1	0.71	0.0	0.1	0.64	0.1	0.2	0.24	0.0	0.1
Rectum *V*_69Gy_ (%)	1.7	1.6	0.04	1.7	1.6	0.05	1.8	1.7	0.15	1.7	1.6	0.04	2.0	1.8
Rectum *V*_64Gy_ (%)	3.3	2.6	0.37	3.2	2.6	0.12	3.3	2.6	0.24	3.2	2.6	0.11	3.5	2.9
Rectum *V*_59Gy_ (%)	5.0	3.5	0.92	4.7	3.5	0.38	4.9	3.5	0.67	4.8	3.5	0.47	5.0	3.9
Penile bulb mean dose (%)	30.8	13.7	≤ 0.001	30.6	14.0	≤ 0.001	27.4	13.3	≤ 0.001	26.2	13.6	≤ 0.001	19.4	12.1
Bladder max dose (%)	106.7	1.2	0.07	106.9	1.1	0.39	108.0	1.5	0.01	108.4	1.5	≤ 0.001	107.2	1.6
Rectum max dose (%)	100.9	8.7	0.04	101.1	8.3	0.09	101.7	9.1	0.47	101.9	9.4	0.72	102.2	8.5

Since all of the clinically‐approved Eclipse plans are 2‐full arc VMAT plans and the majority of clinically approved Ethos plans are 12‐field IMRT plans, it was also of interest to calculate *p*‐values for the same dosimetric indices on the Ethos 2‐full arc VMAT plans, using Ethos 12‐field IMRT plans as the reference. Table 4 provides a side‐by‐side comparison of all dosimetric indices, along with their associated *p*‐values, for the Ethos 12‐field IMRT and 2‐full arc VMAT plans. Dose from the 2‐full arc VMAT was consistently higher than the 12‐field IMRT plan for *D*_max%_ and Bladder maximum dose, while dose from the 12‐field IMRT plan was consistently higher for the Penile Bulb mean dose (*p* ≤ 0.001 for *D*_max%_, Bladder maximum dose, and Penile Bulb mean dose). All other indices were comparable and were found to be clinically acceptable by the radiation oncologist.

**TABLE 4:** Comparison of the normalized Ethos 12‐field IMRT and 2‐field VMAT plans, along with associated *p*‐values, using the normalized Ethos 12‐field IMRT plan as the reference plan

	**Ethos 12‐field IMRT**	**Ethos 2‐field VMAT**	
	**Mean**	**Mean**	***p*‐Value**
*D*_max%_	108.4	109.6	≤0.001
CTV *V*_100%_ (%)	99.9	99.8	0.05
Bladder *V*_79Gy_ (%)	0.0	0.0	N/A
Bladder *V*_74Gy_ (%)	0.3	0.3	0.27
Bladder *V*_69Gy_ (%)	4.5	4.6	0.71
Bladder *V*_64Gy_ (%)	6.8	6.5	0.22
Rectum *V*_74Gy_ (%)	0.0	0.0	0.67
Rectum *V*_69Gy_ (%)	1.7	1.8	0.37
Rectum *V*_64Gy_ (%)	3.2	3.3	0.54
Rectum *V*_59Gy_ (%)	4.7	4.9	0.50
Penile bulb mean dose (%)	30.6	27.4	≤0.001
Bladder max dose (%)	106.9	108.0	≤0.001
Rectum max dose (%)	101.1	101.7	0.09

## 4 DISCUSSION

Lately, automation, machine learning, and AI techniques have been introduced in the radiation therapy community and many systems are commercially available. Soon these techniques are expected to touch all aspects of the radiation therapy workflow: contouring, treatment planning, QA, and treatment delivery with the goal of improving quality, efficiency, and consistency.^31^, ^32^, ^33^, ^34^, ^35^, ^36^, ^37^, ^38^ Varian Ethos is one such commercial system that has introduced automation in different steps of radiation therapy. In this study, we evaluated the efficacy of the Ethos IOE algorithm and its ability to auto‐generate clinically acceptable plans for prostate cancer. Further studies need to be conducted for other anatomical sites.

IOE was able to create both IMRT and VMAT plans efficiently that were comparable to Eclipse clinical plans, which were manually optimized. It may be possible to manually generate a clinically comparable or even superior treatment plan in Eclipse, but at the expense of time and efficiency. It has been reported previously that VMAT plans were comparable to IMRT plans and were the preferred modality due to its efficiency in treatment delivery.^39^, ^40^ However, we noticed that the Ethos VMAT plans were consistently hotter than the Ethos IMRT plans. As a result, the Ethos 12‐field IMRT plans were selected for treatment at our institution. Also, we observed that the Ethos TPS took 13 min to generate a 2‐full arc VMAT plan, compared to 5 min for a 12‐field IMRT plan which could be a desirable feature especially for online adaptive therapy. This demonstrates that the Ethos platform is attempting to arrive at a compromise between plan quality and elapsed planning time. This seems logical as Ethos uses the same geometry and RT intent to create plans in its online adaptive therapy environment, where time is an important factor.

*D*_max%_ was highest for the Ethos 2‐field VMAT plans, when compared to the Eclipse plan and to both Ethos IMRT plans. The observation of Ethos VMAT plans being more dosimetrically inhomogeneous, relative to the IMRT plans, seems to be systematic. Although the Ethos 12‐field IMRT plan was selected for treatment because it met most of the dosimetric criterion, relative to the 9‐field IMRT and 2‐field VMAT plans, the overall results could be deemed clinically insignificant. Furthermore, all Ethos plans were within a clinical range of acceptability, when compared to the Eclipse plan. While OAR dosimetric indices were deemed clinically acceptable by the radiation oncologist, it is possible to reduce manual planning variability by establishing objective templates for all planners that include more dose–volume objectives in the Eclipse TPS. It is also possible to refine automated‐treatment plans by including more dose–volume objectives in the RT intent objectives template.

As demonstrated elsewhere,^7^, ^11^ implementation of AP or KBP algorithms reduced variability among treatment planning quality indices, when compared to human planners. With its IOE, the Ethos TPS is capable of generating dosimetrically acceptable treatment plans and doing so in an efficient time frame.

The IOE seems to create highly modulated plans compared to manual plans resulting in higher MUs, especially in the IMRT plans. Reoptimizing the plans with the same planning directive caused the MUs to change but not significantly. Ethos uses the same geometry and planning directive during adaptive therapy replanning. It was observed that the MUs were higher for an adaptive treatment replan than for the reference plan. One workaround for this was to change the target volume by a miniscule amount and reoptimize the plan. It was observed that, in most instances, the total number of MUs was more comparable to the reference plan MUs when this workaround was executed. More research would be warranted to explain this observation.

One feature that is not available on the current version of Ethos TPS is the ability to overlay and compare DVH indices for multiple plans simultaneously. As mentioned above, Ethos plans needed to be exported into Eclipse to perform multi‐plan intercomparisons. Another desired feature is the ability to apply avoidance sectors in the planning phase. This would be beneficial in generating more optimal plans by avoiding portions of the anatomy (i.e., bilateral prosthetic hip) that would either receive an unnecessary dose or create unwarranted MU increases. The only workaround is to generate a structure and apply a goal in the planning process to limit the radiation to these areas of interest. While Ethos generates multiple plans, the beam geometry consists of fixed, equidistant fields without any beam angle optimization for IMRT plans. It cannot deliver any non‐coplanar fields due to its Halcyon platform limitations. Although Ethos performs auto‐segmentation during the treatment phase, one feature that would be desirable is for auto‐segmentation of structures during the planning phase. These features would allow the Ethos TPS to make use of AI capabilities and become more fully automated.

## 5 CONCLUSION

Varian Ethos TPS can generate multiple treatment plans in an efficient time frame and the quality of the plans could be deemed clinically acceptable when compared to a manually generate treatment plan. Also, while the Ethos 12‐field IMRT plan satisfied most of the clinical goals, the other auto‐generated Ethos plans could be deemed clinically viable. Despite lacking a few desirable features and while still a relatively young product, Ethos TPS has great potential in terms of its ability to incorporate more AI functions into its algorithms, automate planning tasks, and offer multiple treatment plans in an efficient timeframe. This would bring the future of treatment planning one step closer to full automation.

## CONFLICT OF INTEREST

The authors claim no conflicts of interest.

## AUTHOR CONTRIBUTIONS

Shyam Pokharel and Abilio Pacheco were responsible for study design, data acquisition and analysis for this study. Also, both authors contributed equally to the composition and revision of the manuscript. Suzanne Tanner was responsible for patient selection for this study and treatment planning tempate generation in Ethos treatment planning system.

## Supporting information

Supporting information
