# Supplementary material for: Assessment of efficacy in automated plan generation for Varian Ethos intelligent optimization engine
Source: J Appl Clin Med Phys. 2022 Jan 27;23(4):e13539. doi: 10.1002/acm2.13539 (PMC8992949; doi:10.1002/acm2.13539)
Supplement: Supplementary file 1 — Supporting information [file ACM2-23-e13539-s001.docx]

**Assessment of efficacy in automated plan generation for Varian Ethos Intelligent Optimization Engine**

Shyam Pokharel^1, 2^, Abilio Pacheco^1^, Suzanne Tanner^1^

^1^Department of Radiation Oncology, GenesisCare, Naples FL

^2^Department of Radiation Oncology , Lynn Cancer Institute, Boca Raton Regional Hospital, Baptist Health South Florida, Boca Raton, FL

Running title: Assessment of Varian Ethos Intelligent Optimization Engine

Keywords: Adaptive Radiotherapy, Auto planning, Intelligent Optimization Engine, Varian Ethos, Prostate Cancer

Corresponding Author:

Shyam Pokharel, PhD

Lynn Cancer Institute

Boca Raton Regional Hospital

Baptist Health South Florida, Boca Raton, FL

Phone: 239-672-0144

Email: [shyam.pokharel@baptisthealth.net](mailto:shyam.pokharel@baptisthealth.net) or [shyampokharel@gmail.com](mailto:shyampokharel@gmail.com)
